# PEP-1-SOD1 fusion proteins block cardiac myofibroblast activation and angiotensin II-induced collagen production

**DOI:** 10.1186/s12872-015-0103-4

**Published:** 2015-10-07

**Authors:** Li-Guo Tan, Jun-Hui Xiao, Dan-Li Yu, Lei Zhang, Fei Zheng, Ling-Yun Guo, Jian-Ye Yang, Jun-ming Tang, Shi-You Chen, Jia-Ning Wang

**Affiliations:** Institute of Clinical Medicine, Renmin Hospital, Hubei University of Medicine, Shiyan, Hubei 442000 P. R. China; Department of Cardiology, Renmin Hospital, Hubei University of Medicine, Shiyan, Hubei 442000 P. R. China; Department of Emergency, Renmin Hospital, Hubei University of Medicine, Shiyan, Hubei 442000 P. R. China; Departments of Physiology and Pharmacology, University of Georgia, Athens, GA 30622 USA

**Keywords:** Cell-penetrating peptide, Copper, Zinc-superoxide dismutase, Cardiac myofibroblasts, Collagen

## Abstract

**Background:**

Oxidative stress is closely associated with cardiac fibrosis. However, the effect of copper, zinc-superoxide dismutase (SOD1) as a therapeutic agent is limited due to the insufficient transduction. This study was aimed to investigate the effect of PEP-1-SOD1 fusion protein on angiotensin II (ANG II)-induced collagen metabolism in rat cardiac myofibroblasts (MCFs).

**Methods:**

MCFs were pretreated with SOD1 or PEP-1-SOD1 fusion protein for 2 h followed by incubation with ANG II for 24 h. Cell proliferation was measured by Cell Counting Kit-8. Superoxide anion productions were detected by both fluorescent microscopy and Flow Cytometry. MMP-1 and TIMP-1 were determined by ELISA. Intracellular MDA content and SOD activity were examined by commercial assay kits. Protein expression was analyzed by western blotting.

**Results:**

PEP-1-SOD1 fusion protein efficiently transduced into MCF, scavenged intracellular O_2_^−^, decreased intracellular MDA content, increased SOD activity, suppressed ANG II-induced proliferation, reduced expression of TGF-β1, α-SMA, collagen type I and III, restored MMP-1 secretion, and attenuated TIMP-1 secretion.

**Conclusion:**

PEP-1-SOD1 suppressed MCF proliferation and differentiation and reduced production of collagen type I and III. Therefore, PEP-1-SOD1 fusion protein may be a potential novel therapeutic agent for cardiac fibrosis.

**Electronic supplementary material:**

The online version of this article (doi:10.1186/s12872-015-0103-4) contains supplementary material, which is available to authorized users.

## Background

Cardiac fibrosis, characterized by abnormal proliferation of cardiac myofibroblasts (MCF) and excessive deposition of extracellular matrix (ECM), is a leading cause of progressive deterioration of cardiac function and structure in a number of chronic heart diseases including hypertension, coronary heart diseases, and cardiomyopathies. However, no effective therapeutics is currently available. MCFs are the most prevalent cell type in the heart that plays pivotal roles in cardiac fibrosis. Under a number of stimuli such as cytokines (e.g., TGF-β1, IL-1, or endothelin-1), hormones (e.g., angiotensin II (Ang II) or noradrenaline), and mechanical stretch, cardiac fibroblasts can be converted into MCFs exhibiting increased migratory, proliferative, and secretion properties. MCFs are the major source of ECM in the heart with cardiac fibrosis [1].

A growing number of studies have suggested that oxidative stress induced by reactive oxygen species (ROS). (ROS) is associated with cardiac fibrosis. ROS is significantly elevated in hearts of patients and animal models with cardiac fibrosis [2, 3]. Increased ROS triggers a series of cellular events such as differentiation, proliferation, secretion and gene expression. The elevated ROS also increases myofibroblast proliferation and ECM synthesis [4, 5]. Moreover, ROS reduces ECM degradation by regulating the level of matrix metalloproteinases (MMPs) and tissue inhibitor of metalloproteinases (TIMPs) [6]. Superoxide dismutases (SOD) are important antioxidant enzymes that accept an electron from superoxide anion (O^2−^) and H_2_O to generate hydrogen peroxide (H_2_O_2_). Cytoplasm-located SOD1 is the major isoform of SODs in mammalian, and angiotensin II (ANG II)-induced ROS has been found to down-regulate SOD1 activity in rat MCF [7]. These studies suggest that SOD1 is a potential target for preventing cardiac fibrosis. However, exogenous SOD1 cannot be delivered into cells to block oxidative stress because of the lack of specific membrane channel or receptor for SOD1.

PEP-1 ((KETWWETWWTEWSQPKKKRKV), a novel peptide carrier designed by Morris group [8], consists of three domains: a hydrophobic tryptophan rich motif (KETWWETWWTEW), a spacer (SQP), and a hydrophilic lysine-rich domain (KKKRKV). A number of proteins fused with PEP-1 have been efficiently delivered into cells or tissues. Our previous studies have shown that PEP-1-SOD1 fusion protein can be transduced into myocardial and cerebral tissues to protect against oxidative stress induced by ischemia-reperfusion injury [9–11]. In the present study, we found that PEP-1-SOD1 fusion protein, when transduced into rat cardiac myofibroblast, mediates collagen metabolism by blocking ANG II-induced ROS production. Since collagen deposition is a major factor causing fibrosis, PEP-1-SOD1 fusion protein may be a potential therapeutic agent for treating cardiac fibrosis.

## Methods

### Expression and purification of SOD1 and PEP-1-SOD1 fusion protein

SOD1 and PEP-1-SOD1 infusion protein were expressed and purified as previously described [9, 10]. Briefly, two prokaryotic expression plasmids, pET15b-SOD1 and pET15b-PEP-1-SOD1, were constructed with TA-cloning method. The two recombinant plasmids were respectively transformed into E. Coli BL21 (DE3) bacteria (Novagen, USA). Bacteria that have been successfully transformed were grown in 100 ml LB medium containing 100 ug/ml ampicillin at 37 °C to an OD_600_ value of 0.5-1.0 and induced by 0.83 mM isopropyl-β-D-thiogalactoside (IPTG) (Promega, USA) at 25 °C for 12 h. The bacteria were then harvested and lysed by sonication at 4 °C in a binding buffer (5 mM imidazole, 500 mM NaCl, 20 mM Tris–HCl, pH 7.9) for 30 min. The supernatant after centrifugation was loaded onto a Ni^2+^-nitrilotriacetic acid sepharose affinity column (Qiagen, USA) under native conditions. The column was washed with 10 volumes of the binding buffer and 6 volumes of wash buffer (60 mM imidazole, 500 mM NaCl, 20 mM Tris–HCl, pH 7.9), and eluted by an eluting buffer (1 M imidazole, 500 mM NaCl, 20 mM Tris–HCl, pH 7.9). The fusion proteins were collected at the peak of OD_280_. The salts of fusion proteins were removed using a PD-10 column, and the fusion proteins were identified by SDS-PAGE and western blot analysis. The protein concentration was assayed with BCA assay kit (Beyotime Institute of Biotechnology, China).

### Isolation and culture of primary rat cardiac MCFs

All animal procedures were carried out in accordance with Regulations of Good Laboratory Practice for non-clinical laboratory studies of drugs issued by the State Food and Drug Administration of China, and the experimental protocol was approved by the Institutional Animal Care and Use Committee of Hubei University of Medicine. Cardiac ventricular myofibroblasts were obtained from male adult Sprague–Dawley rats. Briefly, adult rats were sacrificed by cervical dislocation under ether anesthesia, and the hearts were excised, rinsed with growth media (Dulbecco’s modified essential media (DMEM) supplemented with 10 % fetal bovine serum, 100 U/ml penicillin G and 100 μg/ml streptomycin) and trimmed of connective tissue and fat. The atria were removed, and the ventricle was cut into small pieces with about 1 mm^3^ size. The ventricle pieces were transplant into 25 cm^2^ culture flasks, allowed to dry briefly and then flooded with 5 ml growth media. Culture flasks were then incubated at 37 °C with 5 % CO_2_ balanced air for 1 week. Cell outgrowths from explanted tissues were digested and passaged when grew to 70 % confluent. The growth media with suspended cells were discarded after 90 min, the fresh growth media was added, and the adhered cells were cardiac myofibroblasts. Cells from passages 1–6 were used in this study. Cells from passage 2 were detected by immunofluorescent staining with von Willebrand factor (VWF), desmin, and vimentin antibodies for identification of endothelial cells, muscle cells and fibroblasts, respectively, as previously described [12].

### Transduction of PEP-1-SOD1 protein into MCF

MCF were grown to confluence on 6-well cell culture plates, and then pretreated with PEP-1-SOD1 fusion proteins or SOD1 proteins at different dosages (0–4 μmol/L) for 0–24 h. Cells were then washed with phosphate-buffered saline (PBS), harvested and lysed for western blot or enzyme activity assay. The SOD activity was measured with a SOD Activity Assay Kit by following the manufacture’s protocols (JianCheng Bioengineering Institute, China). To further confirm transduction of PEP-1-SOD1 fusion proteins, MCF were cultured to confluence in 24-well plates, pretreated with 2 μmol/L PEP-1-SOD1 fusion proteins or SOD1 proteins for 2 h, washed twice with 1 x PBS, and immunostained with anti-His-tag antibody.

### Measurement of malondialdehyde (MDA) content, superoxide dismutase (SOD) activity, and superoxide anion (O_2_^−^)

MDA reflects the peroxide production of cell membrane lipids caused by oxidative stress. MDA content and SOD activity were used as indicators of oxidative damage. The MDA content and SOD activity in MCF were determined with a MDA Assay Kit and SOD Activity Assay Kit (JianCheng Bioengineering Institute, China), respectively. The levels of O_2_^−^ in cells were detected with oxidation-sensitive fluorescent probe dihydroethidium (DHE) (Beyotime Institute of Biotechnology, China) and measured with fluorescent microscopy and flow cytometry as described previously [13].

### MCF proliferation assay

To investigate the effect of PEP-1-SOD1 on MCF proliferation induced by Ang II, MCF were cultured in 96-well cell culture plates, and divided into six groups: vehicle-treated (CTL), SOD1 with vehicle, PEP-1-SOD1 with vehicle, Ang II-treated group, SOD1 with Ang II, and PEP-1-SOD1 with Ang II. The cells were starved in serum-free DMEM for 24 h to mimic the ischemic condition (depletion of nutrients) followed by pretreatment with or without 2 μmol/L of PEP-1-SOD1 or SOD1 for 2 h. The media were replaced with DMEM containing 10 % fetal bovine serum to mimic the reperfusion condition. The cells were then incubated with vehicle or 100 nmol/L Ang II for 24 h. MCF proliferation was assayed with Cell Counting Kit-8 (CCK-8) by following the manufacture’s protocols (Beyotime Institute of Biotechnology, China).

### Immunofluorescent cytochemistry

Fluorescent immunocytochemistry was performed on 24-well cell culture plates as previously described [9]. Briefly, after treatment as described above, cells were washed twice with 1 x PBS, fixed with 1 % paraformaldehyde for 15 min at room temperature, and incubated with rabbit anti-His-tag (diluted 1:100), goat anti-VWF (diluted 1:100), mouse anti-desmin (diluted 1:100), or mouse anti-vimentin antibody (diluted 1:100) (Santa Cruz Biotechnology, USA) at 4 °C overnight. Cells were then incubated with FITC-conjugated second antibodies (diluted 1:250) (Zhongshan Biotechnology, China) at 25 °C for 2 h. Nuclei were stained with DAPI (Sigma, USA). The results were observed under a fluorescent microscope (Nikon, Japan).

### Western blot

MCF were harvested and lysed in a lysis buffer. Western blots were performed as previously described [14]. Equal amount of proteins from each sample were subjected to SDS-PAGE, and then transferred to nitrocellulose membranes. The membranes were blocked with 5 % BSA and incubated at 4 °C overnight with specific primary antibodies: rabbit anti-PCNA (diluted 1:100), rabbit His-tag (diluted 1:100), goat anti-collagen I (diluted 1:200), mouse anti-α-SMA (diluted 1:100), rabbit anti-TGF-β1 (diluted 1:200), goat anti-gp91^phox^ (diluted 1:200) (Santa Cruz Biotechnology, USA), or mouse anti-collagen III (diluted 1:200) (Sigma, USA). The horseradish peroxidase-conjugated second antibodies (diluted 1:5000) (Zhongshan Biotechnology, China) were incubated for 2 h at room temperature. Proteins were detected by ECL detection system.

### MMP-1 and TIMP-1 expression assay

The content of matrix metalloproteinase-1 (MMP-1) or tissue inhibitor of metalloproteinase-1 (TIMP-1) in MCF-conditioned media was examined using rat MMP-1 and TIMP-1 ELISA kits (BioSwamp, China).

### Statistical analyses

All dates are expressed as mean ± SD. Differences between groups were analyzed by one-way analysis of variance. *P* < 0.05 was considered statistically significant difference.

## Results

### Transduction of PEP-1-SOD1 fusion protein into cardiac MCF

SOD1 and PEP-1-SOD1 fusion proteins were successfully expressed and purified as shown in Additional file 1: Figure S1. MCF were isolated and cultured from rat heart. MCF were identified by positive-staining of vimentin and negative staining of endothelial cell and muscle markers vWF and desmin, respectively (Additional file 1: Figure S2).

**Fig. 1:**
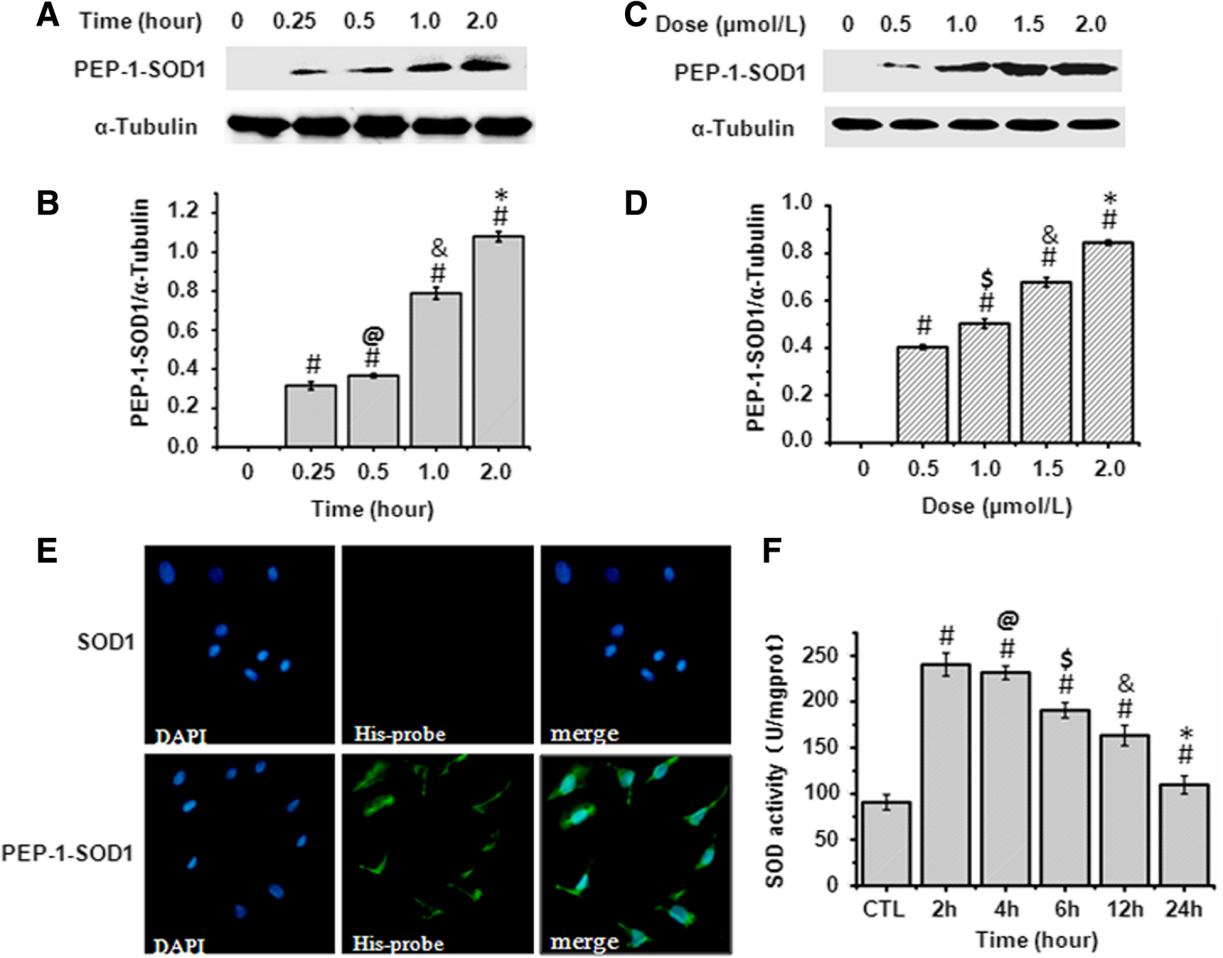
Transduction and enzyme activities of PEP-1-SOD1 fusion protein in MCF. **a**-**d**: Time and dose-dependent transduction of PEP-1-SOD1. **a**, **b**: 2 μM of PEP-1-SOD1 was incubated with cardiac myofibroblast for 0–2 h as indicated, and western blot was performed using anti-His tag antibody (**a**). **b**: Semi-quantitative assay was done in figure A. ^#^ *P* < 0.01 vs. untreated cells (CTL) group; ^@^ *P* > 0.05 vs. 0.25 h group; &*P* < 0.01 vs. 0.5 h group; *P < 0.01 vs. 1.0 h group. (*n* = 5). **c**: 0-2 μM of PEP-1-SOD1 was incubated with cells for 2 h. Western blots were performed the same as in A. **d**: Semi-quantitative assay was done in figure C. ^#^ *P* < 0.01 vs. untreated cells (CTL) group; ^$^ *P* < 0.05 vs. 0.25 h group; &*P* < 0.01 vs. 0.5 h group; *P < 0.01 vs. 1.0 h group. (*n* = 5). **e**: FITC-conjugated PEP-1-SOD1 transduction was detected by immunofluorescent microscopy. **f**: PEP-1-SOD1 exhibited SOD enzymatic activity in MCF. ^#^ *P* < 0.01 vs. untreated cells (CTL) group; ^@^ *P* > 0.05 vs. 2 h group; ^$^ *P* < 0.05 vs. 4 h group; &*P* < 0.05 vs. 6 h group; *P < 0.01 vs. 12 h group. (*n* = 5)

To test the transduction efficiency of PEP-1-SOD1 fusion proteins into cardiac MCF, we used anti-His tag antibody to detect its protein level. As shown in Fig. 1a and c, the fusion protein was observed in MCF 15 min after incubation with 2 μM of PEP-1-SOD1, and the protein level gradually increased when the incubation time increased. However, incubation with SOD1 protein did not result in accumulation of SOD1 in the cells (data not shown). Moreover, the transduction of PEP-1-SOD1 fusion protein, but not SOD1, occurred in a dose-dependent manner (Fig. 1b and d). To further confirm the transduction, PEP-1-SOD1 or SOD1 was conjugated to FITC fluorescein, and the transduction was observed by immunofluorescent microscopy after incubation with the cells. As shown in Fig. 1e, strong green fluorescent signals were observed in cells pretreated with PEP-1-SOD1, but not with SOD1 protein, demonstrating that PEP-1-SOD1, but not SOD1, can enter cardiac myofibroblasts. Importantly, PEP-1-SOD1 is functionally active. As shown in Fig. 1f, SOD1 activity of PEP-1-SOD1 was 2.5-fold higher compared to the untransduced cells, and this high enzyme activity can last for 12–24 h.

### PEP-1-SOD1 fusion protein decreased O_2_^−^ levels and MDA content while enhanced SOD activity

**Fig. 2:**
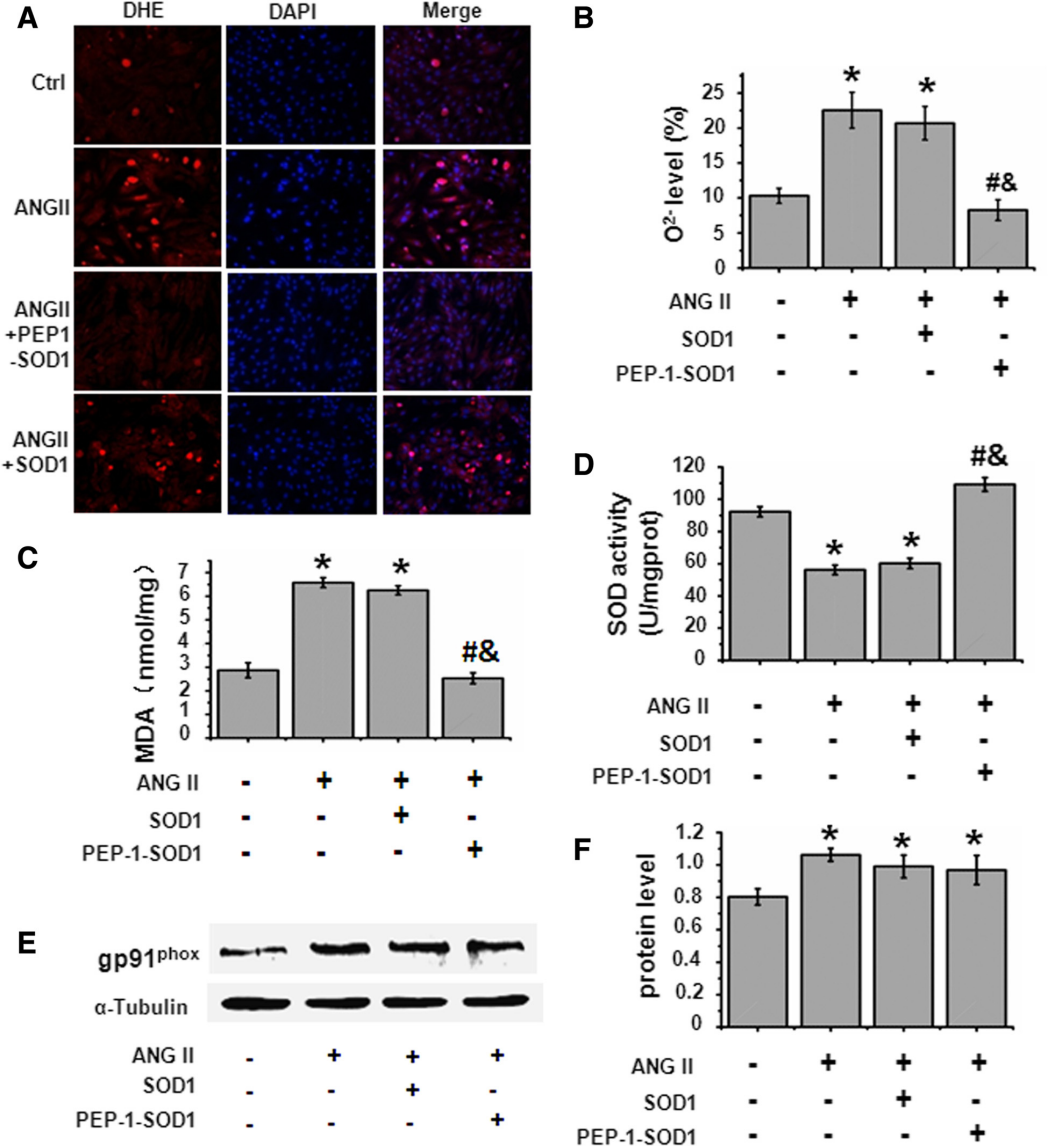
Effect of PEP-1-SOD1 on ANG II-induced ROS in MCF. **a**-**b**: O_2_^−^ levels were detected by fluorescent microscopy (**a**) and quantified by Flow Cytometry (**b**), respectively. Red color was indicated as the changes of O_2_^−^ levels in cells detected with oxidation-sensitive fluorescent probe dihydroethidium (DHE), Blue color was indicated as cell nucleus by DAPI staining. **c**: PEP-1-SOD1, but not SOD1, blocked Ang II-induced increase of MDA content. **d**: PEP-1-SOD1, but not SOD1, restored Ang II-blocked SOD activity. *P < 0.01 vs. Vehicle-treated cells (−), ^#^ *P* < 0.01 vs. Ang II-treated cells, &*P* < 0.01 vs. SOD1-treated cells (*n* = 5). **e**-**f**: gp91^phox^ expression was analyzed by western blot (**e**) and normalized to α-Tubulin (**f**). *P < 0.01 vs. vehicle-treated cells (−) (*n* = 5)

To determine if PEP-1-SOD1 transduction blocks ROS in MCF, we used Ang II to induce ROS production. As shown in Fig. 2a-d, Ang II increased the production of O_2_^−^ and MDA content while decreasing SOD activity in MCF. PEP-1-SOD1 fusion protein pretreatment, however, reduced Ang II-induced O_2_^−^ production and MDA content. PEP-1-SOD1 also enhanced SOD activity in cells. SOD1 pretreatment appeared to have no significant effects on the levels of O_2_^−^, MDA content, or SOD activity mediated by Ang II. These results suggest that PEP-1-SOD1 fusion protein transduction protects MCF from Ang II-induced ROS through the enhanced SOD activity. SOD1 transduction is clearly unable to increase the SOD1 activity in MCF. PEP-1-SOD1 specifically increased SOD1 activity, but did not affect NADPH oxidases because PEP-1-SOD1 did not alter the expression of gp91^phox^, a key subunit of NAPDH oxidases although Ang II also increased gp91^phox^ expression (Fig. 2e-f). As expected, SOD1 transduction had no effect on Ang II-induced gp91^phox^ expression (Fig. 2e-f).

### PEP-1-SOD1 fusion protein attenuated Ang II-induced MCF proliferation

**Fig. 3:**
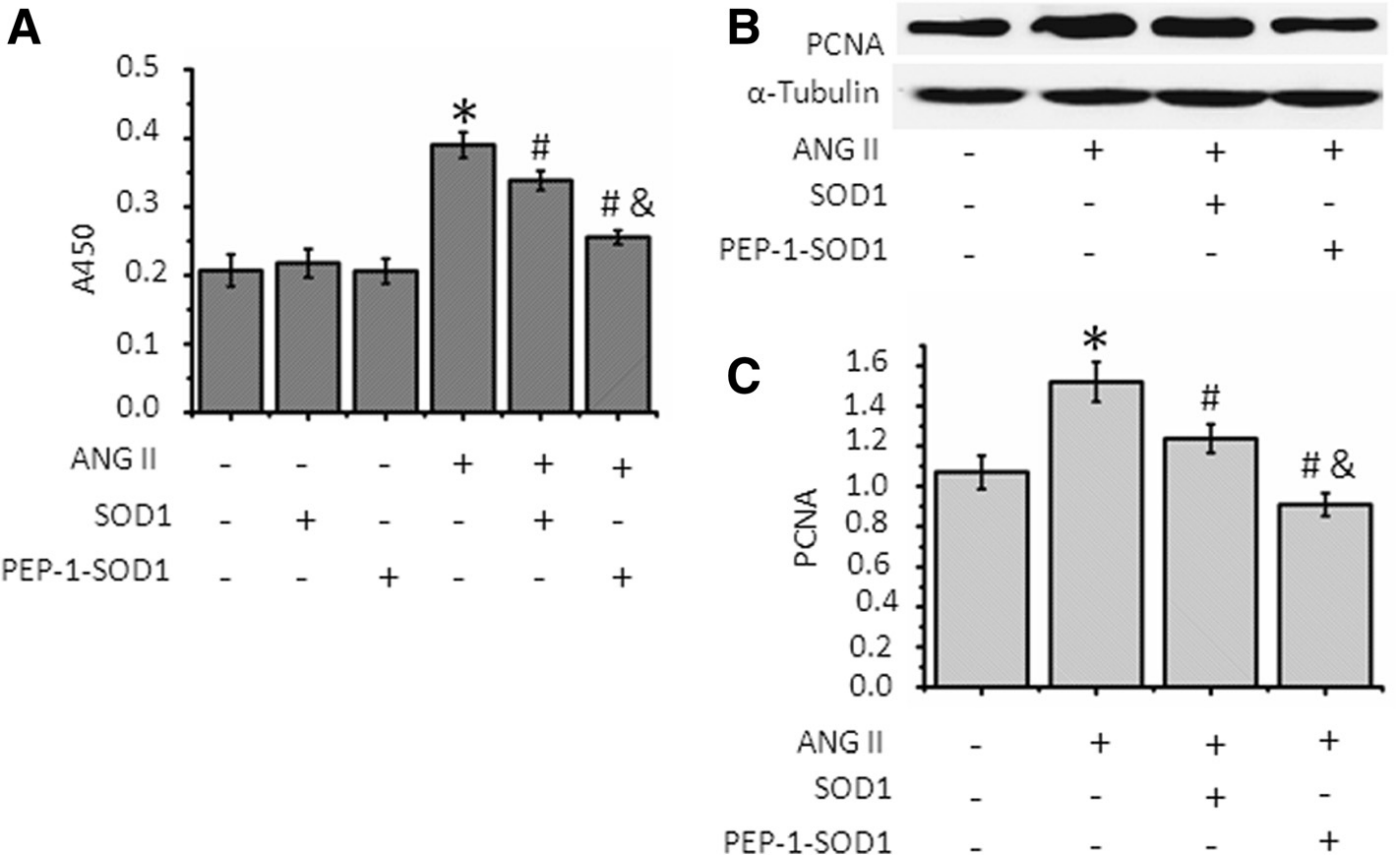
Effect of PEP-1-SOD1 on Ang II-induced MCF proliferation. **a**, MCF proliferation was assayed with CCK-8. **b**-**c**, PCNA expression was measured by western blot (**b**) and quantified by normalizing to α-Tubulin (**c**). *P < 0.01 vs. vehicle-treated cells (−), ^#^ *P* < 0.01 vs. Ang II-treated cells, &*P* < 0.05 vs. SOD1-treated cells (*n* = 5)

Ang II is a potent factor inducing MCF proliferation. We confirmed that Ang II promotes MCF proliferation (Fig. 3a). Pretreatment of SOD1 slightly, but transduction of PEP-1-SOD1 fusion proteins dramatically attenuated the Ang II-induced cell proliferation. Proliferating cell nuclear antigen (PCNA) is a biomarker of cell proliferation. To further determine the effect of PEP-1-SOD1 fusion protein on MCF proliferation, we detected the PCNA expression. Ang II up-regulated PCNA expression (Fig. 3b-c). SOD1 transduction, slightly inhibited, but PEP-1-SOD1 fusion proteins dramatically blocked PCNA expression (Fig. 3b-c). These results indicate that Ang II induces MCF proliferation through increasing ROS production, which can be diminished by the transduction of PEP-1-SOD1.

### PEP-1-SOD1 fusion protein attenuated Ang-II-induced MCF activation

**Fig. 4:**
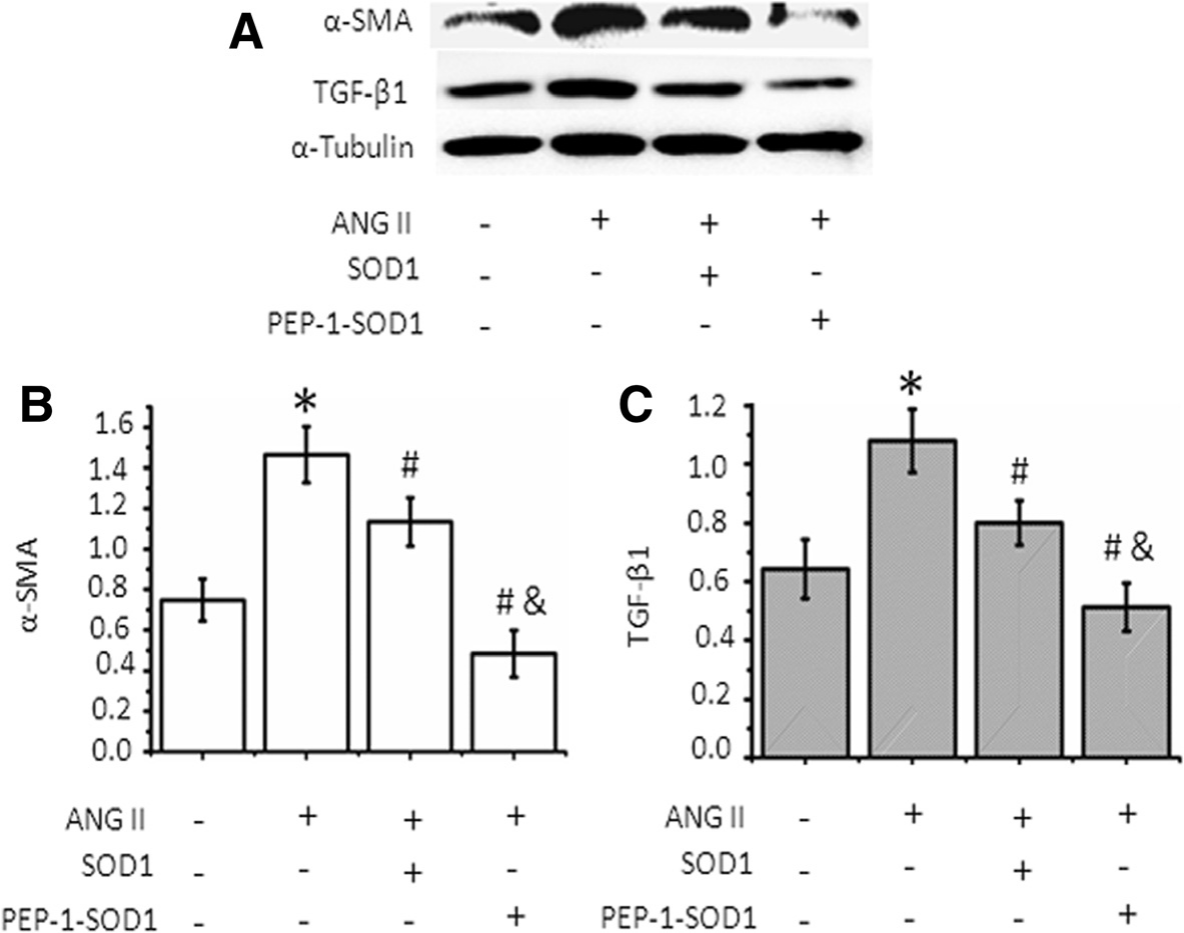
Effect of PEP-1-SOD1 on α-SMA and TGF-β1 protein expression. **a** α-SMA and TGF-β1 protein expression was detected by western blot. **b** Quantification of α-SMA expression by normalizing to α-Tubulin. **c** Quantification of TGF-β1 expression by normalizing to α-Tubulin. *P < 0.01 vs. vehicle-treated cells, ^#^ *P* < 0.01 vs. Ang II-treated cells. &*P* < 0.01 vs. SOD1-transduced cells *n* = 5

Excessive MCF activation accompanied by excessive collagen production is the main mechanism underlying the onset of cardiac fibrosis. Ang II markedly stimulated expression of smooth muscle α-actin (α-SMA) and TGF-β1 in MCF (Fig. 4a-c), indicating a MCF activation. It is known that Ang II induces fibrosis through TGF-β signaling pathway. SOD1 slightly reduced Ang II-induced α-SMA and TGF-β expression. PEP-1-SOD1 infusion protein, however, dramatically blocked Ang II induction of these two genes (Fig. 4). Importantly, PEP-1-SOD1 had a much greater effect as compared to SOD1 (Fig. 4).

### PEP-1-SOD1 blocked Ang-II-induced production of type I and III collagen

**Fig. 5:**
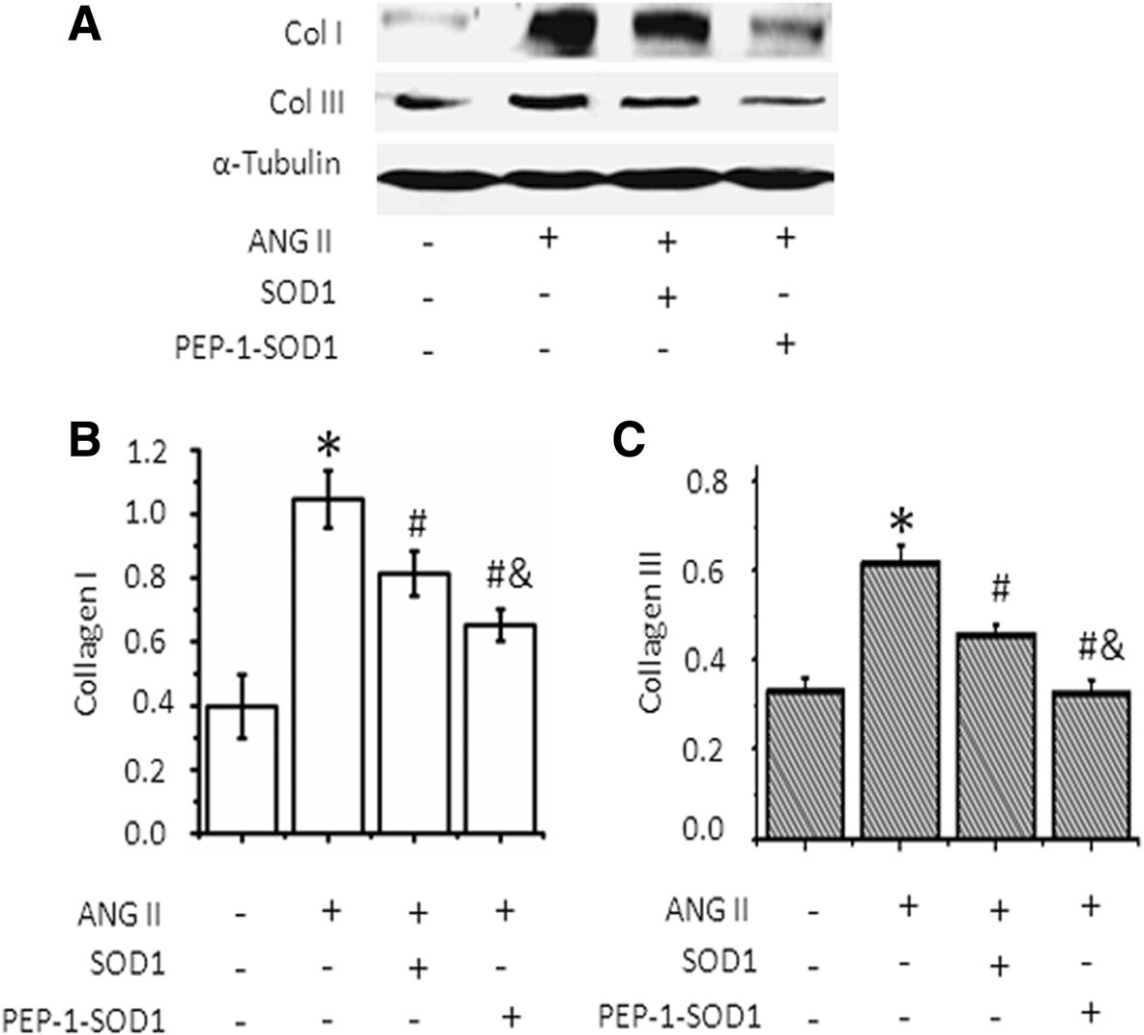
Effect of PEP-1-SOD1 on type I (Col I) and III (Col III) collagen metabolism. **a** Col I and Col III production was analyzed with western blot. **b** Quantification of Col I by normalizing to α-Tubulin. **c** Quantification of Col I by normalizing to α-Tubulin. *P < 0.01 vs. vehicle-treated cells, ^#^ *P* < 0.01 vs. Ang II-treated cells, &*P* < 0.01 vs. SOD1 transduced cells *n* = 5

Activation of MCF leads to production of collagen. To determine if PEP-1-SOD1 fusion plays a role in collagen synthesis, we used Ang II to treat MCF and detected type I (Col I) and III collagen (Col III) protein expression, respectively. As shown in Fig. 5, both the Col I and Col III production were significantly increased over 2.5 fold by Ang II induction. SOD1 pretreatment of MCF marginally, while PEP-1-SOD1 pretreatment dramatically, suppressed Ang-II-induced synthesis of both Col I and Col III (Fig. 5a-c). The effect of PEP-1-SOD1 was much greater compared to that of SOD1. These results demonstrated that delivery of PEP-1-SOD1 can effectively block the excessive production of collagen in activated MCF.

### PEP-1-SOD1 increased Ang-II-mediated blockade of MMP-1 secretion and increase of TIMP-1 production

**Fig. 6:**
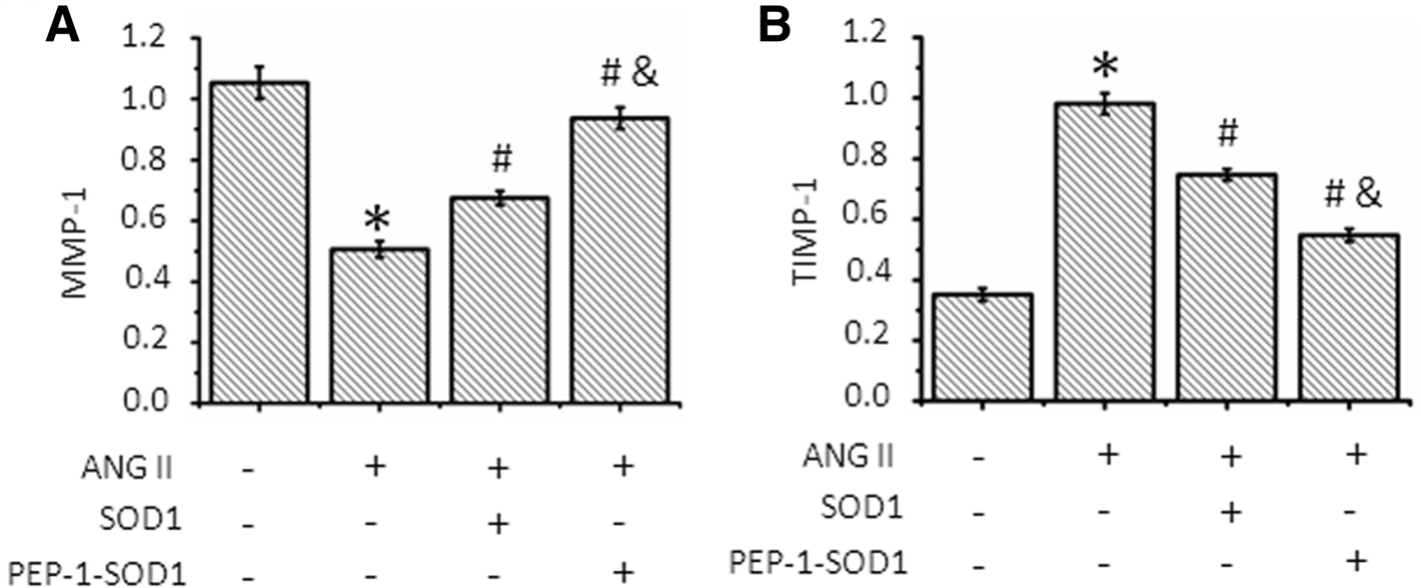
Effect of PEP-1-SOD1 on matrix metalloproteinase-1 (MMP-1) and tissue inhibitors of metalloproteinase 1 (TIMP-1) secretion. The levels of MMP-1 (**a**) and TIMP-1 (**b**) in culture media were examined by ELISA. *P < 0.01 vs. vehicle-treated cells, ^#^ *P* < 0.01 vs. Ang II-treated cells, &*P* < 0.01 vs. SOD1- transduced cells *n* = 5

MMP-1 is a crucial enzyme degrading ECM, and TIMP-1 is a key inhibitor of MMPs. The production of MMP-1 and TIMP-1 is associated with cardiac fibrosis. We found that Ang II reduced the MMP-1 secretion and stimulated production of TIMP-1 in MCF (Fig. 6a-b), consistent with the increased production of Col I and Col III. Pretreatment of SOD1 only marginally reversed Ang II-mediated increase of MMP-1 and reduction of TIMP-1 (Fig. 6a-b). Pretreatment of PEP-1-SOD1, however, almost completely restored Ang II-blocked MMP-1 secretion, and dramatically inhibited Ang II-mediated increase of TIMP-1 secretion (Fig. 6a-b).

## Discussion

Antioxidants have been shown to suppress cardiac fibrosis and improve cardiac function in animal models [15]. Therefore, antioxidant enzymes have been considered as promising therapeutic agents to prevent cardiac fibrosis. SOD1 is a key superoxide dismutase localized in the cytoplasm in mammalian tissues [16], and thus a potential agent for cardiac fibrosis therapy [17]. However, exogenous SOD1 is unable to penetrate into cells or organs to block oxidative stress due to the lack of permeability. Although a number of approaches have been studied for delivering proteins into cells including lipid-, polycationic-, nanoparticle-, and peptide-based methods, these technologies is less effective in pre-clinical or clinical applications because of the poor stability of the complexes formed, the rapid degradation of cargos, or insufficient ability to reach its target. Cell-penetrating peptides are powerful tools used to improve cellular uptake of therapeutic molecules and show bright promise in the clinic application [18]. Our previous studies have demonstrated that PEP-1-SOD1 can be efficiently transduced into cardiomyocytes or neuron cells to prevent these cells from oxidative injury [9, 10, 13]. Our current study demonstrates that PEP-1-SOD1 can also be delivered into MCF and protect MCF from Ang II-induced transdifferentiation. Therefore, PEP-1-SOD1 may be used as a potential therapeutics to treat cardiac fibrosis.

MCF proliferation and activation are the main factors contributing to cardiac fibrosis. Ang II appears to promote both the proliferation and activation because Ang II stimulates MCF growth, PCNA expression, and myofibroblast marker α-SMA expression [19–21]. Although SOD1 transduction only marginally attenuates Ang II function in MCF proliferation and activation, PEP-1-SOD1 exhibits a dramatic effect in blocking Ang II activity. In addition, PEP-1-SOD1 blocks collagen production that is induced by Ang II, suggesting that PEP-1-mediated SOD1 delivery are multi-functional in MCF, i.e., regulating MCF proliferation, differentiation and ECM production. Mechanistically, PEP-1-SOD1 appears to regulate collagen production by modulating MMP1 and TIMP-1 expression because PEP-1-SOD1 reverses Ang II-induced downregulation of MMP1 and upregulation of TIMP-1. Therefore, PEP-1-SOD1 is likely to block TIMP-1 expression, causing increased MMP1 expression. The increased MMP1 then degrades excessive collagen production, leading to the resolution of fibrosis.

Interestingly, although we are unable to detect the SOD1 transduction or activity in MCF, pretreatment with SOD1 exhibits a slight but significant effect on Ang II-induced expression of α-SMA, TGF-β, Col I, Col II, MMP1, and TIMP-1. This is likely due to SOD1 activity in improving ROS status in extracellular environment [22, 23]. It is also possible that SOD1 may slightly affect Ang II interaction with its receptor although the interference does not produce a major impact to Ang II activity. This potential mechanism is obviously a interesting subject for future study.

## Conclusion

Our study show that PEP-1-SOD1 fusion protein can be successfully delivered into MCF to scavenge ROS production and block collagen production, suggesting that PEP-1-SOD1 may be a promising therapeutic agent for treating ROS-mediated cardiac fibrosis.

## Additional file

Additional file 1: Figure S1. Expression and purification of SOD1 and PEP-1-SOD1 fusion proteins. Protein extracts and the purified fusion proteins were resolved in 12% SDS-PAGE (A) and subjected to Western blot analysis with rabbit anti-His-tag antibody (B). Lane 1: pre-stained protein marker; lane 2: total protein extracts for SOD1, lane 3: purified SOD1 proteins; lane 4: total protein extracts for PEP-1-SOD1; lane 5: purified PEP-1-SOD1 fusion proteins. **Figure S2.** Identification of cardiac myofibroblasts. Rat cardiac myofibroblasts were identified by positive staining with anti-vimentin and negative staining with anti-desmin and anti-vWF antibodies. (DOCX 176 kb)

## Abbreviations

PEP-1: Cell-penetrating peptide 1
SOD1: Copper, zinc-superoxide dismutase
ANG II: Angiotensin II
MCF: Cardiac myofibrolast
MMP-1: Matrix metalloproteinases 1
TIMP-1: Tissue inhibitor of metalloproteinases 1
TGF-β1: Transforming growth factor-β1
α-SMA: Smooth muscle α-actin
ECM: Extracellular matrix
ROS: Reactive oxygen species
IPTG: isopropyl-β-D-thiogalactoside
DMEM: Dulbecco’s modified essential media
VWF: Von Willebrand factor
MDA: Malondialdehyde
O^2−^: Superoxide anion
PCNA: Proliferating cell nuclear antigen
gp91phox: Cytochrome b-245, beta polypeptide
Col I: Collagen type I
Col III: Collagen type III
