# Supplementary material for: PEP-1-SOD1 fusion proteins block cardiac myofibroblast activation and angiotensin II-induced collagen production
Source: BMC Cardiovasc Disord. 2015 Oct 7;15:116. doi: 10.1186/s12872-015-0103-4 (PMC4597385; doi:10.1186/s12872-015-0103-4)
Supplement: Additional file 1: Figure S1. — Expression and purification of SOD1 and PEP-1-SOD1 fusion proteins. Protein extracts and the purified fusion proteins were resolved in 12% SDS-PAGE (A) and subjected to Western blot analysis with rabbit anti-His-tag antibody (B). Lane 1: pre-stained protein marker; lane 2: total protein extracts for SOD1, lane 3: purified SOD1 proteins; lane 4: total protein extracts for PEP-1-SOD1; lane 5: purified PEP-1-SOD1 fusion proteins. Figure S2. Identification of cardiac myofibroblasts. Rat cardiac myofibroblasts were identified by positive staining with anti-vimentin and negative staining with anti-desmin and anti-vWF antibodies. (DOCX 176 kb) [file 12872_2015_103_MOESM1_ESM.docx]

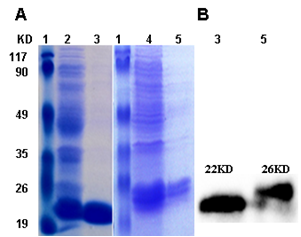


**Figure S1: Expression and purification of SOD1 and PEP-1-SOD1 fusion proteins.** Protein extracts and the purified fusion proteins were resolved in 12% SDS-PAGE (A) and subjected to Western blot analysis with rabbit anti-His-tag antibody (B). Lane 1: pre-stained protein marker; lane 2: total protein extracts for SOD1, lane 3: purified SOD1 proteins; lane 4: total protein extracts for PEP-1-SOD1; lane 5: purified PEP-1-SOD1 fusion proteins.


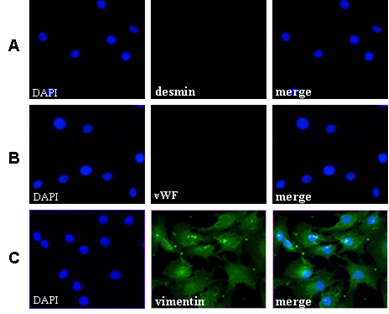


**Figure S2: Identification of cardiac myofibroblasts.** Rat cardiac myofibroblasts were identified by positive staining with anti-vimentin and negative staining with anti-desmin and anti-vWF antibodies.
